# Observed and Predicted Risk of Breast Cancer Death in Randomized Trials on Breast Cancer Screening

**DOI:** 10.1371/journal.pone.0154113

**Published:** 2016-04-21

**Authors:** Philippe Autier, Mathieu Boniol, Michel Smans, Richard Sullivan, Peter Boyle

**Affiliations:** 1 Strathclyde Institute of Global Public Health at iPRI, Lyon, France; 2 International Prevention Research Institute (iPRI), Lyon, France; 3 Institute of Cancer Policy, Kings Health Partners Cancer Centre, Bermondsey Wing, Guy’s Campus, London, United Kingdom; University of North Carolina School of Medicine, UNITED STATES

## Abstract

**Background:**

The role of breast screening in breast cancer mortality declines is debated. Screening impacts cancer mortality through decreasing the number of advanced cancers with poor diagnosis, while cancer treatment works through decreasing the case-fatality rate. Hence, reductions in cancer death rates thanks to screening should directly reflect reductions in advanced cancer rates. We verified whether in breast screening trials, the observed reductions in the risk of breast cancer death could be predicted from reductions of advanced breast cancer rates.

**Patients and Methods:**

The Greater New York Health Insurance Plan trial (HIP) is the only breast screening trial that reported stage-specific cancer fatality for the screening and for the control group separately. The Swedish Two-County trial (TCT)) reported size-specific fatalities for cancer patients in both screening and control groups. We computed predicted numbers of breast cancer deaths, from which we calculated predicted relative risks (RR) and (95% confidence intervals). The Age trial in England performed its own calculations of predicted relative risk.

**Results:**

The observed and predicted RR of breast cancer death were 0.72 (0.56–0.94) and 0.98 (0.77–1.24) in the HIP trial, and 0.79 (0.78–1.01) and 0.90 (0.80–1.01) in the Age trial. In the TCT, the observed RR was 0.73 (0.62–0.87), while the predicted RR was 0.89 (0.75–1.05) if overdiagnosis was assumed to be negligible and 0.83 (0.70–0.97) if extra cancers were excluded.

**Conclusions:**

In breast screening trials, factors other than screening have contributed to reductions in the risk of breast cancer death most probably by reducing the fatality of advanced cancers in screening groups. These factors were the better management of breast cancer patients and the underreporting of breast cancer as the underlying cause of death. Breast screening trials should publish stage-specific fatalities observed in each group.

## Introduction

In 1985, publications of the Greater New-York Health Insurance Plan and of the Two-County Trial suggested that 2 to 4 rounds of breast screening could reduce the risk of breast cancer death by 30% [1, 2]. Following these publications, mammography screening expanded rapidly in the USA, Sweden, Finland, the United Kingdom, and in the Netherlands. Three other randomized trials conducted in the 1980-90s in Sweden also obtained risk reductions ranging from 10 to 22% [3]. Two randomized trials conducted in Canada obtained no reduction in the risk of breast cancer death with mammography screening [4, 5].

Meta-analyses of these trials have suggested that a breast cancer mortality reduction of about 20% could be expected if at least 70% of women aged 40 to 74 years attended two to five rounds of mammography screening [6]. However, studies in Europe that compared changes in breast cancer mortality between areas with similar economic level and access to treatment, but with early or with late (i.e., 10 to 15 years later) implementation of mammography screening found no difference in changes of breast cancer mortality rates over time [7–9]. The progressive introduction of mammography screening in Swedish counties had no impact on breast cancer mortality trends at county level [10]. In the USA, participation to screening ranging from 40 to 90% did not affect rates of breast death after 30 years of screening [11]. No association was found between the timing and magnitude of declining trends in breast cancer mortality and the timing of mammography screening implementation in the various States of the USA [12]. This apparent absence of impact of mammography screening on breast cancer mortality was in sharp contrast with studies that clearly showed quicker and steeper reductions in the risk of cervical and colorectal cancers in areas where screening is widespread, compared to areas where screening is not common [13–15].

How can such contrast between results of meta-analyses and of population studies on breast screening be most logically explained? We hypothesize that randomized trials overestimated reductions in risks of breast cancer death associated with screening. The hypothesis can be tested in a simple way. Advanced cancers are highly fatal and screening reduces cancer mortality through decreasing numbers of patients diagnosed with an advanced cancer. Hence, the reduction in the risk of cancer death observed in a trial should be equivalent to the reduction of the risk of cancer deaths associated with the lower rate of advanced cancer in the screening group as compared to the control group (i.e., the predicted reduction). If the observed risk reduction is greater than the predicted risk reduction, then it is likely that factors other than screening have diminished the fatality of advanced cancers in the screening group.

The objective of this study is to test this hypothesis. For didactic purpose, we first tested the hypothesis for the Prostate Lung Colorectal and Ovarian (PLCO) trial on colorectal cancer (CRC) screening with flexible sigmoidoscopy that reported the relevant data [16].

## Methods

### Cancer fatality

In a randomised trial, cancer fatality is the proportion of cancer patients who, from randomisation until the end of follow-up, die because of their cancer. Cancer fatality can be readily deduced from trial data (Table 1). The early stage cancers are usually small, local cancers, and the advanced cancers are cancers with regional extension (e.g., in regional lymph nodes) or with metastases in distant organs. At end of follow-up, for each category of cancer stage, the numbers of cancer patients and of cancer deaths are totalised. Stage specific fatality can be deduce by dividing numbers of cancer deaths by numbers of cancer patients (i.e., f = d/n). Conversely, the number of cancer deaths in each stage category can be derived from the number and the cancer-specific fatality of cancer patients (d = n*f).

**Table 1:** Typical results of randomised trials testing the efficacy of interventions aiming at reducing the risk of cancer death.

	Intervention group			Control group		
	No. cancer patients	Fatality (%)	No. cancer deaths	No. cancer patients	Fatality (%)	No. cancer deaths
Early cancers	n_ei_	f_ei_	d_ei_	n_ec_	f_ec_	d_ec_
Advanced cancers	n_ai_	f_ai_	d_ai_	n_ac_	f_ac_	d_ac_
Total	N_i_	-	D_i_	N_c_	-	D_c_

Keys: “e” stands for early cancer; “a” stands for advanced cancer”; “i” stands for intervention; “c” stands for control.

Assuming that numbers of subjects randomised in the intervention and control group are identical, an efficient screening method would reduce the number of advanced cancers in the intervention group (n_ai_<n_ac_), but their fatality would remain the same (f_ai_ = f_ac_). As a consequence there would be fewer cancer deaths due to late diagnosis in the intervention than in the control group (d_ai_<d_ac_), and the relative risk computed as Di/Dc would be smaller than 1.0. In contrast, an efficient treatment would reduce the fatality of advanced cancer in the intervention group (f_ai_<f_ac_), but their numbers would remain the same (n_ai_ = n_ac_). As a consequence among subjects with advanced cancer, there would be fewer cancer deaths in the intervention than in the control group (d_ai_<d_ac_), and the relative risk computed as Di/Dc would be smaller than 1.0. It follows that numbers of cancers deaths and relative risks observed in a trial can be predicted from knowledge of the number of cancer patients in each stage category and the fatality specific to each stage category.

### Literature search

We performed a systematic search on PubMed for articles on breast screening trials. We also searched reference lists in major reviews [17–19] and in publications on breast screening trials we already had. We retrieved all articles reporting original data of these trials. Three of us (PA, MB, MS) read the articles with looking for cancer-specific fatality data reported by categories of size or lymph-node status or stage. For the sake of finding comparable data on screening for other cancers, we performed a similar literature search for colorectal cancer. Fatality could be reported as a proportion of cancer patients who died because of the cancer or as a survival statistics, which is the reverse of fatality. It could be reported as a measure of the risk (e.g., the hazard rate) to die from a large (more advanced) cancer compared to the risk to die from a small (less advanced) cancer. We also looked for breast screening trials that performed their own assessment of predicted numbers of breast cancer deaths.

### Statistical analysis

The statistical methods we used were the same as those used by Morrison [20] and by Tabar et al, 1995 [21]. When fatality rates were reported for the screening and control group separately, we computed the predicted numbers of cancer deaths in the screening group using the size-, or stage-, or lymph-node specific fatality rates of the control group. We then computed the predicted relative risk and 95% confidence interval using the predicted number of cancer deaths in the screening group and the number of observed cancer deaths in the control group. When fatality rates were calculated after pooling breast cancer patients found in the screening and the control group, we computed the predicted numbers of cancer deaths in the screening and in the control group using the size-, or stage-, or lymph-node specific fatality rates reported for both groups. We then computed the predicted relative risk and 95% confidence interval using the predicted numbers of cancer deaths in the screening and in the control group.

For the Two-County trial, two articles reported numbers of breast cancers by size categories diagnosed in women 40 to 74 years of age during the intervention period [22, 23]. Another article reported the 11-year size-specific crude hazard rates of breast cancer death in women 40 to 69 years of both screening and control groups [24]. Hazard rates represented the risk of breast death of women diagnosed with a cancer 10 mm size or more relative to the risk of breast cancer death of women diagnosed with a small (1–9 mm) breast cancer. The transformation of hazard rates into fatality (in percent) can be done using the cumulative mortality formula displayed in the Appendix of Tabar et al, 1995 [21], i.e.,

[1−exp(hazard ratio*In(1−0.052))]

The value 0.052 is the 11-year fatality rate for the reference category of cancers 1–9 mm size, that was derived from Figure 7 of Tabar et al, 1992 [22].

As an example, we first applied the statistical method on data reported by the PLCO trial on flexible sigmoidoscopy [16].

## Results

### The PLCO colorectal cancer screening trial

The PLCO trial reported an observed relative risk of colorectal cancer death of 0.74 (95% CI: 0.63 to 0.87) associated with screening with flexible sigmoidoscopy [16](Table 2). There were 225 fewer stage II-IV CRCs in the screening than in the control group. There were also fewer CRCs in the screening than in the control group because sigmoidoscopy allows the removal of adenomas. Stage-specific fatality rates in the screening and in the control group were comparable, but for the carcinoid tumours, probably because screening could detect slow-growing carcinoid tumours. We computed the predicted number of colorectal cancer death in the screening group by multiplying cancer numbers in each stage category of the screening group by the fatality of corresponding categories of the control group. We obtained a predicted relative risk of 0.76 that was close to the observed relative risk of 0.74.

**Table 2:** Reported and predicted risk of colorectal cancer (CRC) death in the PLCO trial*.

	Screening group (N = 77,445 subjects)			Control group (N = 77,455 subjects)			
Stage	No. CRC	No. CRC deaths	Fatality (%)	No. CRC	No. CRC deaths	Fatality (%)	Predicted no. of CRC deaths in the screening group †
I	334	20	0.060	407	21	0.052	17
II	240	26	0.108	309	33	0.107	26
III	241	70	0.290	328	102	0.311	75
IV	140	113	0.807	209	163	0.780	109
Carcinoid	32	3	0.094	9	4	0.444	14
Unknown	25	20	0.800	25	18	0.720	18
Total	1012	252		1287	341		259
	Observed relative risk = 252/341 = 0.74 (95% CI:0.63–0.87)						Predicted relative risk = 259/341 = 0.76 (95% CI: 0.65–0.89)

This is the Table 2 legend.

* Data are from Schoen et al, 2012 but the predicted number of colorectal cancer deaths.

^†^ Equal to the no. of CRC in the screened group multiplied by the fatality in the control group.

### Breast cancer screening trials

The Greater New-York Health Insurance Plan (HIP) trial and the Two-County Trial (TCT) reported relevant data. Other studies also computed the predicted relative risks of breast cancer death for these trials [20, 21]. The Age trial performed a comparison of observed and of predicted breast cancer mortality but did not report fatality rates observed during the trial [25]. We therefore just summarized the key findings of this trial. For the Malmö, Stockholm and Goteborg trials, we found no article that reported on size, lymph-node or stage-specific fatality.

### The Greater New-York Health Insurance Plan (HIP) trial

After 7 year-follow-up, the HIP trial reported 97 breast cancer deaths in the screening group for 131 in the control group, resulting in an observed relative risk of 0.72 (95% CI: 0.56–0.94) (Table 3)[26].

**Table 3:** Reported and predicted breast cancer deaths in the HIP trial*.

	Screening group (N = 30,239)				Control group (N = 30,756)		
	No. BCs	Observed BC deaths	7-year fatality (%)	Predicted BC deaths †	No. BCs	Observed BC deaths	7-year fatality (%)
Lymph node negative	170	31	0.185	44	130	33	0.256
1+ positive lymph nodes	102	48	0.475	66	121	79	0.650
Lymph node status not known	27	16	0.592	17	34	21	0.620
In situ	38	1	0.026	5	24	3	0.126
Totals	337	97		131	309	136	
Relative risk		0.72 ‡		0.98 §			
95% CI		0.56 to 0.94		0.77 to 1.24			

This is the Table 3 legend.

BC: breast cancer

*Data on BC number and on fatality from Table 12 of Shapiro et al, 1977.

^†^ By multiplying numbers of BC of screening group by the fatality in the control group.

^‡^ Observed relative risk.

^§^ Predicted relative risk.

At first sight the trial fulfilled the objective to reduce breast cancer deaths associated with advanced cancer because there were 31 (i.e., 79–48) fewer breast cancer deaths in women with positive lymph-node in the screening as compared to the control group. However, there were only 19 (i.e., 121–102) fewer women with positive lymph-node in the screening as compared to the control group. So, the number of breast cancer deaths among lymph-node positive women had reduced more than the number of women with positive lymph-node. This result was possible because the 7-year fatality in the screening group was 27% lower (i.e., 0.475/0.650) than in the control group. Similarly, the 7-year fatality of lymph-node negative women was 28% lower (i.e., 0.185/0.256) in the screening than in the control group. As a consequence, when 7-year fatality rates observed in the control group were used for predicting breast cancer deaths in the screening group, there were 66 predicted instead of 48 observed breast cancer deaths among lymph-node positive women, and 44 predicted instead of 33 observed breast cancer deaths in lymph-node negative women.

Overall, using fatality rates reported for the control group, we predicted 5 fewer breast cancers associated with screening, equating to a predicted relative risk of 0.98 (95% CI: 0.77–1.24). There were 14 (5%) more invasive breast cancers in the screening than in the control group, and the predicted relative risk computed after exclusion of extra cancers was similar. Hence, reductions in breast cancer deaths in the HIP trial were essentially the consequence of reductions in fatality rates that were due to factors other than screening.

Morrison who had direct access to HIP data [27] used 10-year fatality rates of breast cancers in the control group for predicting reduction in breast cancer deaths in the screening group. The prediction was 8 fewer breast cancer deaths, equating to a predicted relative risk of 0.94 (95% CI: 0.75–1.19).

### The Two-County trial

Size-specific numbers of breast cancer reported by the Two-county trial after 11 years of follow-up are displayed in columns (1) and (2) of Table 4 [22]. In column (3), we corrected the numbers of breast cancer in the control group for the imbalance in group size. There were 85 fewer breast cancer deaths in the screening than in the control group and the relative risk of breast cancer death was 0.73 (95% CI: 0.62–0.87).

**Table 4:** Computations of predicted numbers of breast cancer deaths in the Two-County trial.

	No. Invasive breast cancer patients *			Breast cancer patients fatality during the trial		Predicted no. invasive breast cancer patients		Exclusion of extra cancers in the screening group			
	Screening group (N = 77,080 women)	Control group (N = 55,585 women)	Control group, corrected for size (N = 77,080 women)	Crude hazard ratio for breast cancer death	Fatality (%)	Screening group	Control group	Difference screening-control group	Screening group without extra cancers		Predicted no. of breast cancer deaths in screening group without extra cancers
Column no.:	(1)	(2)	(3)	(4)	(5)	(6) = (1)*(5)	(7) = (3)*(5)	(8) = (3)-(1)	(9)	(10) = (9)	(11) = (10)*(5)
Invasive breast cancer size (mm):											
1–9	245	50	69	1.00	0.052	13	4	*176*	**= 69+39**	108	6
10–14	305	107	147	2.03	0.103	31	15	*158*	**= 147+73**	220	23
15–19	248	143	197	2.56	0.128	32	25	*51*	**= 197+23**	220	28
20–29	258	216	297	6.33	0.287	74	85	**-39**	= 258	258	74
30–49	124	143	197	13.01	0.501	62	98	**-73**	= 124	124	62
50+	71	68	94	27.89	0.774	55	72	**-23**	= 71	71	55
Total no. cancer cases	1251	727	1003					**248**		1001	
Number of breat cancer deaths:	234	232	319			267	299				248
Computation of the relative risk of breast cancer death:	234/319 =					267/299 =					248/299 =
Relative risk of breast cancer death:	0.73†					0.89					0.83
95% CI:	0.62–0.87					0.75–1.05					0.70–0.97

CI: confidence interval; PY: person-year;

* After 11-year follow-up, there were 833552 PYs of follow-up in the screening group and 606241 PYs in the control group (Tabar et al, RSNA, 1992).

^†^ The reported crude relative risk in Tabar et al, RSNA, 1992 was 0.71 (95% CI: 0.59–0.88).

(1)(2) Data from Tabar et al, RSNA, 1992, cancers of women 40 to 74 years of age at trial start invited to screening and control women before first screening.

(3) Obtained by multiplying patient numbers in column (2) by 1.375 (= 833552 PYs/606241 PYs).

(4) From Duffy et al, 1991.

(5) Results of a Cox's proportional hazard regression model (see method section).

A comparison of columns (1) and (3) shows that there were 135 less invasive cancers having a size of 20 mm or more in the screening than in the control group, and 385 more invasive cancers 1 to 19 mm size in the screening than in the control group(column (8) of Table 4). A part of extra cancers 1 to 19 mm size proceeds from the advance in diagnosis of cancer that would have been larger in the absence of screening (i.e., lead time effect) and another part are screen-detected cancers that would probably have never become clinical (i.e., length time effect), thus representing overdiagnosis.

According to the Two-county trial investigators, only 1% of in situ and invasive breast cancer found in the screening group of this trial would represent overdiagnosis [28]. So, considering that practically no extra invasive breast cancer would be overdiagnosed, and using the hazard ratios reported in Duffy et al (1991),[24] we first computed the predicted 11-year fatalities (Column (5)), and then the numbers of predicted cancer deaths in the screening and in the control group (columns (6) and (7)). The predicted relative risk was 0.89 (95% CI: 0.75–1.05). This predicted relative risk suggests that the reduction in the risk of breast of cancer death due to down-staging was 11%, and that the remaining reduction of 16% (i.e., (1–0.73)-0.11) would be due to factors other than screening.

As a sensitivity analysis, we performed computations considering that all extra cancers represented overdiagnosis. To this end, we redistributed the 135 cancers 20 mm or more deemed to have been detected earlier thanks to screening (column (9)) in size-specific categories of cancers less than 20 mm of the control group. We performed these transfers of cancer cases with keeping the ranking of cancers across size categories. For instance, the 108 cancers 1 to 9 mm in column (10) were the sum of the 69 cancers 1 to 9 mm of the control group (column (3)) and of the 39 fewer cancers 20 to 29 mm in the screening group (column (8)). The predicted relative risk was 0.83 (95% CI: 0.70–0.97).

Using the same fatality data and statistical formula we used, the Two-county trial investigators computed predicted relative risks of breast cancer death for women 40 to 49 and 50 to 74 years, equating to a predicted relative risks of 0.76 (95% CI: 0.64–0.89) for women 40 to 74 years (S1 Table)[21]. This predicted relative risk is smaller than the ones we obtained but still at a distance from the relative risk of 0.69 reported by the Two-county trial investigators [21]. The dissimilarity between our predicted relative risk and that of Two-county trial investigators proceeds from the total number of breast cancer in the control group (not adjusted for the difference in group size): 1041 in the S1 Table for 729 in Table 3. The difference of 312 cancers in the control group corresponds to the addition by the Two-county trial investigators of 266 invasive and 46 in situ breast cancers found at first screening of the control group that took place in years following the end of the intervention period [22, 23]. The 266 invasive cancers included 73 cancers of 20 mm or more, which boosted the numbers of predicted breast cancer deaths in the control group, and led to a predicted relative risk of 0.76 much smaller than the one of 0.89 we obtained. The article of Tabar et al (1995)[21] does not allude to the addition of breast cancers found at first screening of control women to breast cancers found in this group during the screening period.

### The Age trial

After a mean follow-up of 10.7 years, the observed relative risk of breast cancer death in the Age trial was 0.83 (95% CI: 0.66–1.04)[29]. The cumulative incidence of breast cancers 20 mm or more was 3.2 per 1,000 women in the screening group for 3.6 per 1,000 women in the control group [30]. For women who were part of the Age trial for at least 10 years, the observed relative risk of breast cancer death was 0.79 (95% CI: 0.60–1.06)[29].

The Age trial investigators computed predicted relative risks using tumour size, grade, and lymph-node status for a 10-year period. Predicted relative risks were 0.90 (95% CI: 0.80–1.01) when the Nottingham Profile Index was used and 0.89 (95% CI: 0.78–1.01) when the Two-County trial procedure was used [21, 25]. There were 8% more invasive breast cancers in the screening than in the control group, and a predicted relative risk computed after exclusion of extra cancers was likely to be similar.

## Discussion

Our analysis found that in the randomized trial on colorectal cancer screening with flexible sigmoidoscopy, the observed and predicted relative risks were equivalent, indicating that the reduction in colorectal cancer mortality obtained by the trial was entirely due to stage-shift associated with screening. In contrast, in breast screening trials, the relative risks reported in publications were substantially smaller than the predicted relative risks, which suggests that a substantial part of reductions in breast cancer mortality obtained by these trials were due to factors other than reductions in the incidence rate of advanced breast cancer associated with screening. No other breast screening trial reported data allowing the comparison of observed and of predicted risk reductions. However, a recent study [31] computed an overall predicted relative risk of breast cancer death in women 50–74 years of age using the evidence that the mean cumulative rates of advanced cancer in the screening groups of all breast screening trials was on average 15% lower than in control groups [32]. The Birnbaum et al. study estimated that reductions in rates of advanced cancer would translate into a mean 10% reduction in the risk of breast cancer death. This 10% reduction is in sharp contrast with meta-analyses of breast screening trials have reported relative reductions of 23% in the risk of breast cancer death in women aged 50 years or more [19].

Published data of the HIP trial provide clues about the non-screening factors implicated in this trial. The fatality of breast cancer patients with none or with one or more positive lymph nodes was 27% lower in the screening than in the control group (Table 2) [33]. In the 1960s, no efficient chemotherapeutic adjuvant or therapeutic regimen existed, and the HIP cause of death committee was totally blinded as to the randomization status of women [34]. The more plausible explanation for the lower fatality proceeds from data showing first that although women who did not participate to screening represented 35% of women in the screening group, only 25% (i.e., 74/299) of breast cases and 29% (i.e., 28/96) of breast cancer deaths occurred in these non-participants (S2 Table). Second that the breast cancer fatality in women invited to screening but who did not attend screening was lower than the fatality of interval cancers and of cancers in the control group (S2 Table). These data in non-participants are clearly anomalous. In the Two-County trial, 27% of breast cancer deaths occurred in the 15% of women who did not participate to screening [22] and an abundant literature documents that breast cancers diagnosed in women not attending screening are more advanced and have a fatality rate 1.5 to 2 times greater than that of interval cancers or of cancers in control women [24, 35–39]. Investigations of characteristics of women included in the HIP trial revealed that the tracing of women who refused screening had been arduous, which may have led to information gaps on cancer occurrence and causes of death for many of these women [40]. So, the breast cancer mortality reduction in the HIP trial was most probably due to the failure to register a substantial number of breast cancer deaths in non-participants.

In the Two-county trial, the observed relative risk was 0.73 (0.62–0.87), while the predicted relative risk was 0.89 (0.75–1.05) if, as suggested by investigators, [28] overdiagnosis was assumed to represent 1% of all cancers. The predicted relative risk was 0.83 (0.70–0.97) if extra cancers were excluded. Similarly to the HIP trial, could the size-specific fatalities in the Two-county trial have been lower in the screening than in the control group? The Two-county trial never reported size-specific fatalities for the screening and for the control group separately. It is therefore impossible to formally establish that in this trial, the size-specific fatalities of the large breast cancers were smaller in the screening than in the control group. But an indirect proof exists. If fatalities of large cancers were actually smaller in the screening than in the control group, then the reported mean fatalities (noted “f_am_”) should be greater than the actual (unknown) fatalities in the screening group, and smaller than the actual (unknown) fatalities in the control group (i.e., f_ai_ <f_am_<f_ac_ in Table 1). As a consequence, computations of predicted breast cancer deaths in the screening group using the mean fatalities (i.e., n_ai_*f_am_) will end up in a predicted number of breast cancer deaths greater than the observed number. Conversely, computations of predicted breast cancer deaths in the control group using the mean fatalities (i.e., n_ac_*f_am_) will end up in a predicted number of breast cancer deaths smaller than the observed number. As a matter of fact, in Table 4, the reported and predicted numbers of breast cancer deaths in the screening group were 234 and 267 respectively, whilst in the control group these numbers were 319 and 299, respectively. These numerical relationships accredit the hypothesis that in the Two-county trial, size-specific fatalities in the screening group were lower than in the control group.

The other Swedish trials and the UK Age trial did not report breast-cancer specific fatality according to size, stage, or lymph node status for the screening and the control group separately. Moss et al did not provide a firm explanation for discrepancies they found in the UK Age trial [29]. A legitimate concern is thus that in all trials that found a reduced risk of breast cancer death associated with screening, the stage (or size or lymph-node) specific fatality of breast cancer women was lower in the screening than in the control group.

The quest for factors that may have contributed to a lower fatality in screening groups needs to consider the “left-to-nature” design adopted by all trials that found reduced risk of breast cancer death associated with screening. Typically, parallel group randomized trials first recruit a group of eligible subjects that are informed on trial objectives, on potential health benefits and probable side effects. Subjects agreeing to participate must first sign an informed consent form after which they are randomized in a screening or in a control group. This typical procedure was used in the Canadian trials [4, 5]. In left-to-nature trials, only women invited to participate in breast screening knew they were part of a clinical trial. Women allocated to control groups were never contacted, did not sign an informed consent and were completely ignorant they were part of a trial. All the follow-up of control women was purely administrative, with linkage between the trial and the cancer registries, and also linkage with the cause of death registry if causes of death were those reported on death certificates. Health professionals could thus know or detect which women were invited to screening. In contrast, health professionals could not identify which women in the population were allocated to control groups.

Size-specific fatality is derived from numbers of breast cancer patients in each size category found during the intervention period and from numbers of breast cancer deaths observed in each size category (Table 1). A first question is whether the assessment of cancer size (or stage, or lymph-node status) could have differed between the screening and the control group, with the consequence that compared to the control group, breast cancer patients of the screening group would have been systematically classified in larger size categories. This hypothesis assumes that compared to the control group, reductions in large size cancers in the screening group were underestimated. This hypothesis is unlikely because cancer size is measured during the histological examination of the surgically removed breast tissues. The same laboratories analysed surgical materials of women invited and not invited to screening, and there is no reason to believe that the histological analyses could have been different for women invited and not invited to screening.

In the Two-county trial, causes of death were established by local endpoint committees, and in the UK Age trial, death certificates were used. The absence of blinding of health professionals may have caused underreporting of breast cancer as the underlying cause of death of women invited to screening, which could have biased results in favour of screening. This hypothesis is supported by knowledge that relative risks of breast cancer death in the Two-county trial were always lower when causes of death were derived from death certificates than from the local endpoint committee [41, 42]. But death certificates are also prone to reporting bias, as shown by an intervention study on skin screening in Germany where the absence of blinding of doctors ended in the underreporting of melanoma as the underlying cause of death on death certificates [43, 44].

The lower fatality in the screening group may also have been due to the better medical management of women invited to screening (i.e., a performance bias). Better management means quicker referral to work-up procedures of women found with a breast lump, better access to specialized care, and more intense therapy.

In conclusion, our study indicates that in three randomized trials for which relevant data were available, the actual ability of mammography screening to decrease the risk of breast cancer death had been overestimated. Breast screening trials should make available the cancer-specific survival or fatality rates by stage or by size for the screening and control groups, separately.

## Supporting Information

S1 STROBE Checklist Checklist of items that should be included in reports of observational studies. (DOC)

S1 Table Predicted numbers of breast cancer deaths in the two-County trial reported in Tabar et al, Cancer, 1995, table 6. * Data in italicized letters were not reported table 6 of Tabar et al, 1995.[1]. † Includes invasive and in situ cancers.
(DOCX)

S2 Table Reported invasive breast cancer patients and deaths in the HIP trial. BC: breast cancer. *Data from Table 9 of Shapiro et al, 1977 [1].
(DOCX)
