# Supplementary material for: Observed and Predicted Risk of Breast Cancer Death in Randomized Trials on Breast Cancer Screening
Source: PLoS One. 2016 Apr 21;11(4):e0154113. doi: 10.1371/journal.pone.0154113 (PMC4839680; doi:10.1371/journal.pone.0154113)
Supplement: S1 Table — * Data in italicized letters were not reported table 6 of Tabar et al, 1995.[1]. † Includes invasive and in situ cancers. (DOCX) [file pone.0154113.s002.docx]

**Supplementary materials to the article “Observed and predicted risk of breast cancer death in randomized trials on breast cancer screening” by P. Autier, M. Boniol, M. Smans, R. Sullivan, and P. Boyle.**

**S1 Table S1. Predicted numbers of breast cancer deaths in the two-County trial reported in Tabar et al, 1995, [1] table 6.***

| **Age** | **Study group** | **Total predicted deaths (10-year)** | **No. breast cancer †** | **No. Women** | **Relative risk** | **95% CI** |
| --- | --- | --- | --- | --- | --- | --- |
| 40-49 | Intervention | 51.01 | 256 | 19844 |  |  |
|  |  |  |  |  | 0.96 | 0.73 to 1.27 |
|  | Control | 41.68 | 162 | 15604 |  |  |
| 50-74 | Intervention | 226.23 | 1170 | 57296 |  |  |
|  |  |  |  |  | 0.71 | 0.63 to 0.81 |
|  | Control | 224.10 | 879 | 40381 |  |  |
| *40-74* | *Intervention* | *277.24* | *1426* | *77140* |  |  |
|  |  |  |  |  | *0.76* | *0.64 to 0.89* |
|  | *Control* | *265.78* | *1041* | *55985* |  |  |
| * Data in italicized letters were not reported table 6 of Tabar et al, 1995.[1] | | | |  |  |  |
| † Includes invasive and in situ cancers. | | |  |  |  |  |

**References**

1. Tabar L, Fagerberg G, Chen HH, Duffy SW, Smart CR, Gad A, et al. Efficacy of breast cancer screening by age. New results from the Swedish Two-County Trial. Cancer. 1995;75(10): 2507-2517.
