# Supplementary material for: Observed and Predicted Risk of Breast Cancer Death in Randomized Trials on Breast Cancer Screening
Source: PLoS One. 2016 Apr 21;11(4):e0154113. doi: 10.1371/journal.pone.0154113 (PMC4839680; doi:10.1371/journal.pone.0154113)
Supplement: S2 Table — BC: breast cancer. *Data from Table 9 of Shapiro et al, 1977 [1]. (DOCX) [file pone.0154113.s003.docx]

**Supplementary materials to the article “Observed and predicted risk of breast cancer death in randomized trials on breast cancer screening” by P. Autier, M. Boniol, M. Smans, R. Sullivan, and P. Boyle.**

**S2 Table. Reported invasive breast cancer cases and deaths in the HIP trial.***

|  | No. BCs | % of BC of screening group | 7-year fatality rate (%) | No. BC deaths |
| --- | --- | --- | --- | --- |
| Intervention group (N=30,239) | 299 |  | 0.322 | 96 |
| Screen-detected | 132 | 0.44 | 0.213 | 28 |
| Interval | 93 | 0.31 | 0.424 | 39 |
| Non participants | 74 | 0.25 | 0.385 | 28 |
| Control group (N=30,756) | 285 |  | 0.467 | 133 |
| Relative risk |  |  |  | 0.72 |
| 95% CI |  |  |  | 0.56 to 0.94 |
| BC: breast cancer | | | | |
| *Data from Table 9 of Shapiro et al, 1977. [1] | | | | |

**References**

1. Shapiro S. Evidence on screening for breast cancer from a randomized trial. Cancer. 1977;39(6 Suppl): 2772-2782.
